# Construction of a Female Sterility Maintaining System Based on a Novel Mutation of the *MEL2* Gene

**DOI:** 10.1186/s12284-024-00688-x

**Published:** 2024-02-04

**Authors:** Xia Wang, Shuting Yuan, Changjian Wang, Wei Yan, Gang Xie, Cuifang Wang, Shijun Qiu, Jianxin Wu, Xing Wang Deng, Chunjue Xu, Xiaoyan Tang

**Affiliations:** 1 Guangdong Provincial Key Laboratory of Biotechnology for Plant Development, School of Life Sciences, South China Normal University, 510631 Guangzhou, China; 2 Shenzhen Institute of Molecular Crop Design, 518107 Shenzhen, China; 3 School of Advanced Agricultural Sciences, Peking University, 100871 Beijing, China

**Keywords:** Female sterility maintaining system, Female sterile line, Hybrid rice seed production, MEL2

## Abstract

**Background:**

Hybrid rice has significant yield advantage and stress tolerance compared with inbred rice. However, production of hybrid rice seeds requires extensive manual labors. Currently, hybrid rice seeds are produced by crosspollination of male sterile lines by fertile paternal lines. Because seeds from paternal lines can contaminate the hybrid seeds, mechanized production by mixed-seeding and mixed-harvesting is difficult. This problem can be solved if the paternal line is female sterile.

**Results:**

Here we identified a female infertile mutant named *h569* carrying a novel mutation (A_1106_G) in the *MEL2* gene that was previously reported to regulate meiosis entry both in male and female organs. *h569* mutant is female infertile but male normal, suggesting that MEL2 regulates meiosis entry in male and female organs through distinct pathways. The *MEL2* gene and *h569* mutant gave us tools to construct female sterility maintaining systems that can be used for propagation of female sterile lines. We connected the wild-type *MEL2* gene with pollen-killer gene *ZmAA1* and seed-marker gene *DsRed2* in one T-DNA cassette and transformed it into ZZH1607, a widely used restorer line. Transgenic line carrying a single transgene inserted in an intergenic region was selected to cross with *h569* mutant. F_2_ progeny carrying homozygous A_1106_G mutation and hemizygous transgene displayed 1:1 segregation of fertile and infertile pollen grains and 1:1 segregation of fluorescent and non-fluorescent seeds upon self-fertilization. All of the non-fluorescent seeds generated female infertile plants, while the fluorescent seeds generated fertile plants that reproduced in the way as their previous generation.

**Conclusions:**

These results indicated that the female sterility maintaining system constructed in the study can be used to breed and propagate paternal lines that are female infertile. The application of this system will enable mechanized production of hybrid rice seed by using the mixed-seeding and mixed harvesting approach, which will significantly reduce the cost in hybrid rice seed production.

**Supplementary Information:**

The online version contains supplementary material available at 10.1186/s12284-024-00688-x.

## Background

The breeding and large-scale planting of hybrid rice has contributed significantly to the food supply worldwide (Ma and Yuan 2015). Currently, two technologies, the “three-line system” and the “two-line system”, are used for hybrid rice production (Liao et al. 2021). The “three-line system” consists of a cytoplasmic male sterile (CMS) line that carries a cytotoxic CMS gene, a maintainer line that is genetically identical to the CMS line but lacks the CMS gene, and a restorer line that carries a different nuclear genome with *Rf* gene(s) capable of suppressing the CMS gene function (Chen and Liu 2014). Propagation of the CMS line is mediated by crosspollination of the CMS line by the maintainer line, while production of the hybrid seeds is through crosspollination of the CMS line by the restorer line (Chen and Liu 2014). The “three-line system” has been adopted for hybrid rice production since 1970s, and it occupied 4.9 million hectares in China in 2015 (Bai et al. 2018). The “two-line system” uses a photoperiod/thermo-sensitive genic male sterile (PTGMS) line and a paternal line of different nuclear genome (Cheng et al. 2007). The male sterility phenotype of PTGMS lines is controlled by a recessive nuclear gene that is sensitive to changes of day-length and/or environmental temperature (Peng et al. 2022). Propagation of the PTGMS line is through self-pollination under environmental conditions restoring its male fertility, while production of hybrid seeds is through crosspollination by the paternal line under environmental conditions suppressing the male fertility of the PTGMS line (Peng et al. 2022). The “two-line system” has been adopted for rice production since 1990s, and it occupies ~ 4.6 million hectares in China (Bai et al. 2018).

Current method for hybrid rice seed production is mostly by growing the two parental lines in alternative rows, usually 6–12 rows of the MS line and 1–2 rows of the paternal line (Tang et al. 2020). To facilitate crosspollination, human assistance is required to shake the paternal plants so the pollen grains can fly up into the air (Tang et al. 2020). Common practices include shaking the paternal plants with a long stick or by two people who pull a rope tightly over the rice plants and walk fast on both sides of the field (Maruyama et al. 1991). It usually requires ~ 3–4 back-and-forth walks during the pollen shedding period every day and continues for 7–10 days to achieve high crosspollination (Li et al. 2013). If the seed production field is large, a helicopter or an unmanned aerial vehicle can be used to assist crosspollination (Li et al. 2013). Despite of the human assistance, the distance between the MS line and paternal line is still a barrier to high crosspollination (Maruyama et al. 1991). After crosspollination, paternal lines are manually removed before seed-setting, or the two lines are harvested separately to avoid mixing of the paternal seeds into the hybrid seeds (Tang et al. 2020). These practices are tedious and highly rely on manual operations, which hinder the wide adoption of hybrid rice, particularly in countries where labor cost is high (Tang et al. 2020).

To reduce the seed production cost, people have attempted several methods that are amenable to mixed seeding of parental lines and mechanization production. One method is by using parental lines of different seed sizes. For example, a MS line of small grain is mix-seeded with a paternal line of large grain. The resulting F_1_ hybrid seeds are small and can be separated from the paternal seeds using a sieve (Yu et al. 2007; Tang et al. 2020). Another method is by using parental lines of different husk colors (He et al. 2001). For example, a MS line with a pale yellow husk is mix-seeded with a paternal line of purple hull. The resulting F_1_ hybrid seeds have pale yellow husk and can be separated from the paternal seeds using a color sorting machine (He et al. 2001). Parental lines of different herbicide sensitivity have also been tested for mixed seeding and mechanical harvest (Fu et al. 2010; Zhang et al. 2010). The MS line is herbicide resistant, while the paternal line is herbicide sensitive. After pollination, the field is sprayed with herbicide to kill the paternal line. Despite all the attempts, none of the methods has been adopted for commercial production of hybrid seeds, because all of them have intrinsic drawbacks such as low efficiency and difficulty to avoid contamination of the paternal line (Tang et al. 2020). At present, a method that is amenable to mixed-seeding and mixed-harvesting production of hybrid rice seeds is still highly desirable.

A paternal line with normal male fertility but female sterile is considered ideal for mixed-seeding and mixed-harvesting production (Maruyama et al. 1991), because it can pollinate MS lines to produce hybrid seeds but itself cannot produce seeds. Therefore, it can be mix-seeded with the MS line and does not need to be removed after pollination. However, the female sterile line cannot reproduce itself, so the key problem is how to propagate the female sterile line in a large scale.

In this study, we isolated a novel female sterile rice mutant controlled by a recessive nuclear gene. This mutant is completely normal in vegetative development and male fertility. We report the development of a transgenic system that can be used to propagate the female sterile lines based on this mutation.

## Results

### Isolation and Morphological Analysis of the *h569 *Mutant

By screening the M_2_ lines derived from ethyl methanesulfonate (EMS)-treated seeds of *indica* rice Huanghuazhan (HHZ) (Chen et al. 2014), we identified a M_2_ line named h569 consisting of 2 plants of complete sterility and 10 plants of normal fertility. Seeds from the fertile plants were harvested, and 20 M_3_ seeds from each M_2_ plant were planted again. Seven groups showed segregation of 4–6 sterile individuals and the remaining fertile individuals. These observations suggested that the sterility was likely controlled by a recessive mutation. We named the sterile mutants *h569* following the name of the M_2_ line.

**Fig. 1:**
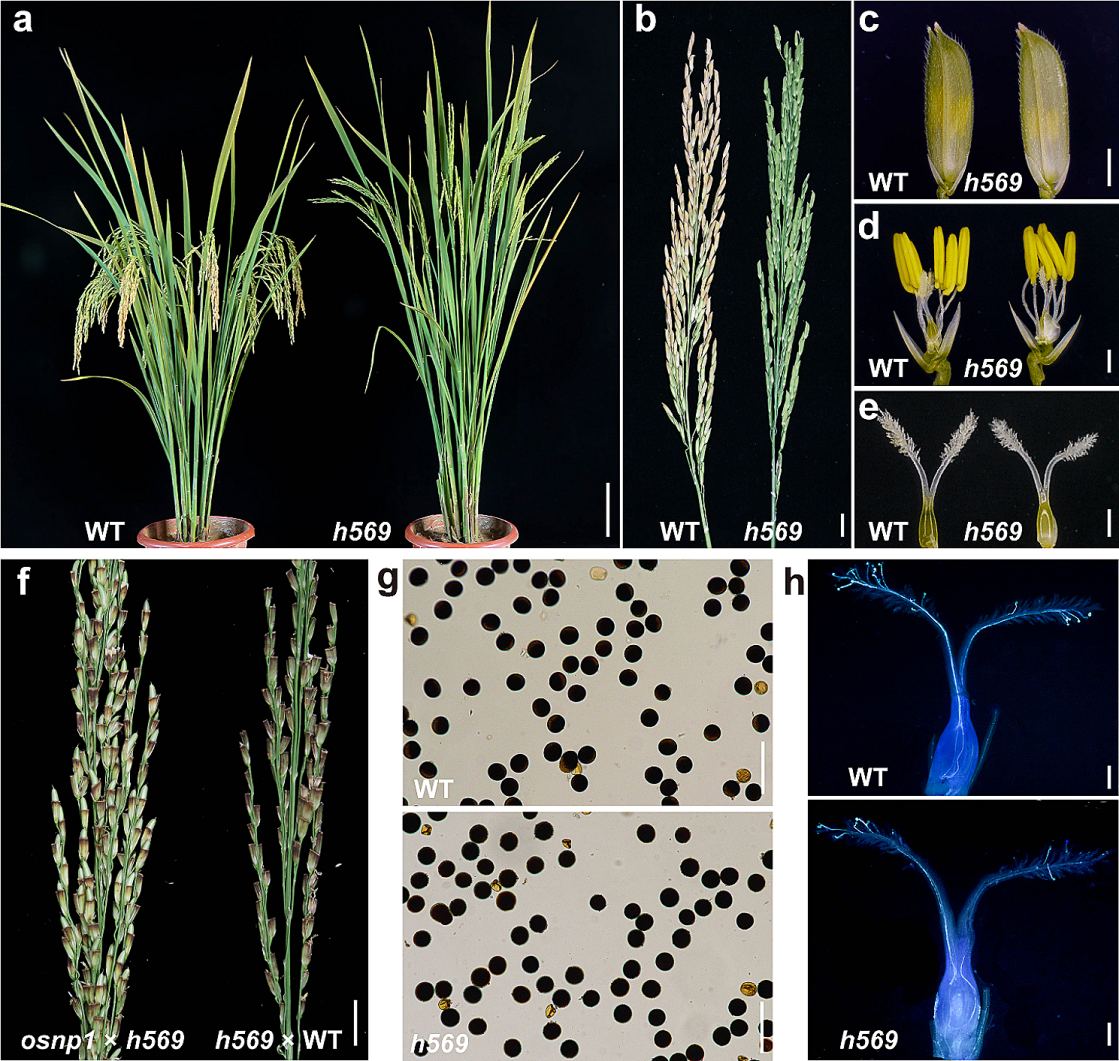
Phenotype of *h569* mutant. **a**, Wild-type HHZ (WT) and *h569* plants; bar = 10 cm. **b**, Panicles of WT and *h569* mutant; bar = 1 cm. **c**, Spikelets of WT and *h569* mutant; bar = 1 mm. **d**, Spikelets of WT and *h569* mutant with the palea and lemma removed; bar = 1 mm. **e**, Pistils of WT and *h569* mutant; bar = 1 mm. **f**, Seed-setting of *osnp1* and *h569* plants 20 d after pollination (*osnp1* pollinated with the *h569* pollen, and *h569* pollinated with the WT pollen); bar = 1 cm. **g**, Pollen grains of WT HHZ and *h569* with I_2_-KI staining; bar = 100 μm. **h**, Pollen tube growth in the pistils of HHZ and *h569* mutant; bar = 1 mm

**Fig. 2:**
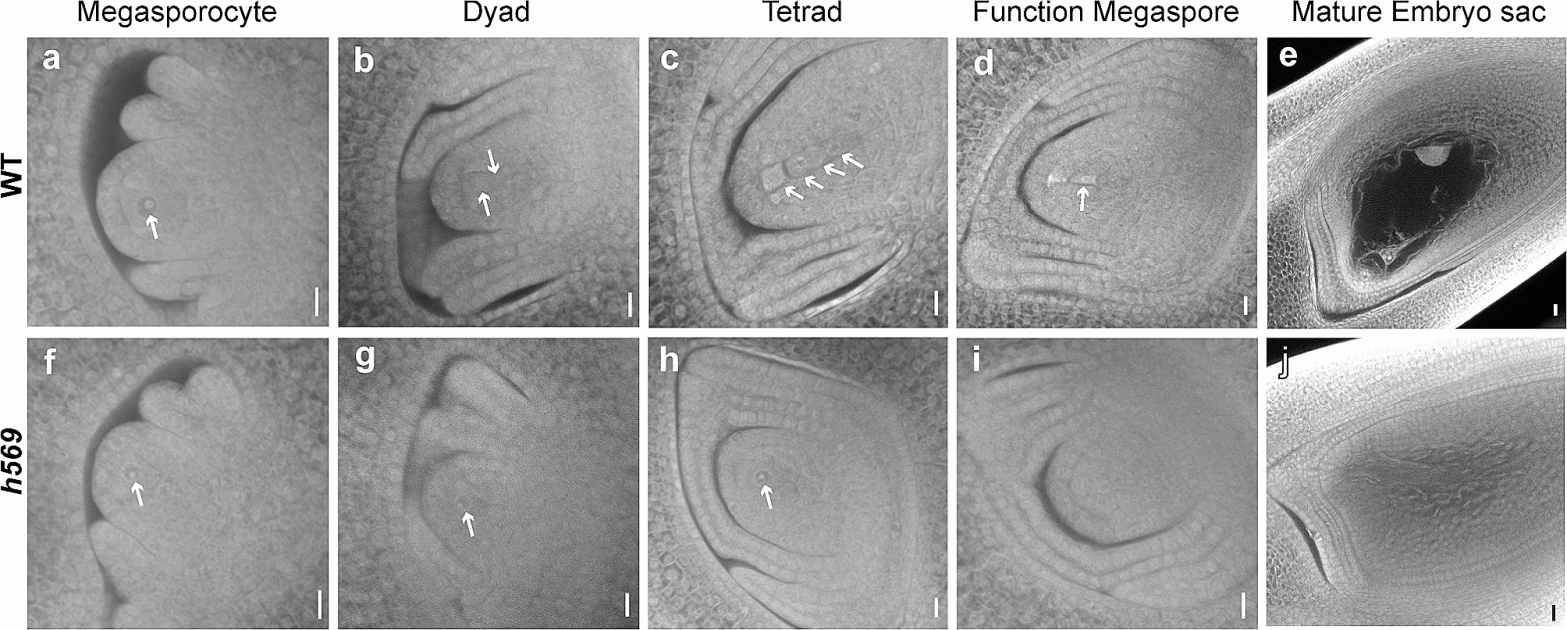
Embryo sac development in the WT and *h569* mutant. **a–e**, Embryo sac development in the WT. Arrows show the megaspore mother cell, dyad, tetrad, functional megaspore, and mature embryo sac. **f–j**, Embryo sac development in *h569* mutant. Pistils at the same developmental stage as the WT were analyzed. Bars = 10 μm

The vegetative development, inflorescence, spikelet, anther, and pistil of *h569* mutant all looked normal (Fig. 1a–e). I_2_-KI staining of mature pollen grains showed no difference between the mutant plants and wild-type (WT) (Fig. 1g). To determine the cause of sterility, the mutant plants were used as the recipient of the WT HHZ pollen or as the pollen donor to pollinate the male sterile line controlled by recessive nuclear gene *osnp1* (Chang et al. 2016a). No seed-setting was found in the mutant plants after they were pollinated by WT HHZ (Fig. 1f). However, when used as pollen donor, *h569* mutant induced normal seed-setting in the MS line *osnp1* (Fig. 1f). These results indicated that the mutant plants were female sterile. To understand how *h569* mutant impairs female fertility, pollen germination and pollen tube growth were examined in *h569* mutant after it was pollinated with the WT HHZ pollen. Pollen germinated normally and grew all the way to the micropyle in *h569* mutant as it did in the WT HHZ (Fig. 1h), suggesting that the failure in seed-setting was probably due to absence of fertilization. The *h569* mutant pistil looked normal outside (Fig. 1e). Thus, we asked whether the lack of fertility was caused by abnormal ovule or embryo sac development. The development of WT and *h569* mutant ovule and embryo sac was observed with a confocal microscope. Both WT and *h569* mutant showed normal ovary wall and integument, but the development of embryo sac was different between them. In the WT, the megaspore mother cell (MMC) was clearly visible, and it went through meiotic divisions to produce a tetrad (Fig. 2a–c). Subsequently, the three daughter cells near the micropyle end gradually degenerated and disappeared, and the remaining one developed into a functional megaspore (Fig. 2d), which in turn underwent three rounds of mitosis to form a mature embryo sac (Fig. 2e). Unlike the WT, *h569* mutant displayed abnormal embryo sac development. MMCs could be formed in the mutant, but they could not go through meiosis (Fig. 2f). No dyad, tetrad, functional megaspore and mature embryo sac were found in *h569* mutant (Fig. 2g–j). We examined 50 WT and 50 *h569* pistils at the mature embryo sac stage. All the WT pistils showed normal embryo sac, whereas all of *h569* pistils showed a filled ovule without a sign of embryo sac development. These observations indicated that the abnormal development of embryo sac in *h569* led to its female infertility.

To determine the genetic control of the mutant phenotype, *h569* mutant was backcrossed with WT HHZ. All F_1_ plants exhibited normal fertility. The F_2_ population showed an approximate 3:1 segregation of fertile and sterile plants (211:72, χ^2^_3:1_ = 0.029 < χ^2^_0.05_, _1_=3.84; Table S1), indicating that the female sterile phenotype was caused by a recessive gene.

The sterility of *h569* mutant was highly stable. During propagation of more mutant materials for experiments in different seasons of different years, we found that most of the mutant plants exhibited no seed-setting. Occasionally, we found a few infertile plants bearing one or a few seeds. To investigate this in more detail, we examined five segregating populations at the F_3_ generation. A total of 114 mutant plants were examined. Among them, 63 plants did not bear any seeds, 23 plants set one seed, 14 plants set two seeds, 7 plants set three seeds, 5 plants set four seeds, and 2 plants set five seeds. The average population seed-setting rate of *h569* mutant was 0.03%, with the highest seed-setting rate of 0.2% on one individual mutant plant (Fig. S1). Plants derived from these seeds were all infertile. These results indicated that a very small number of mutant pistils were fertile.

### Identification of the Causal Mutation for ***h569*** Mutant

**Fig. 3:**
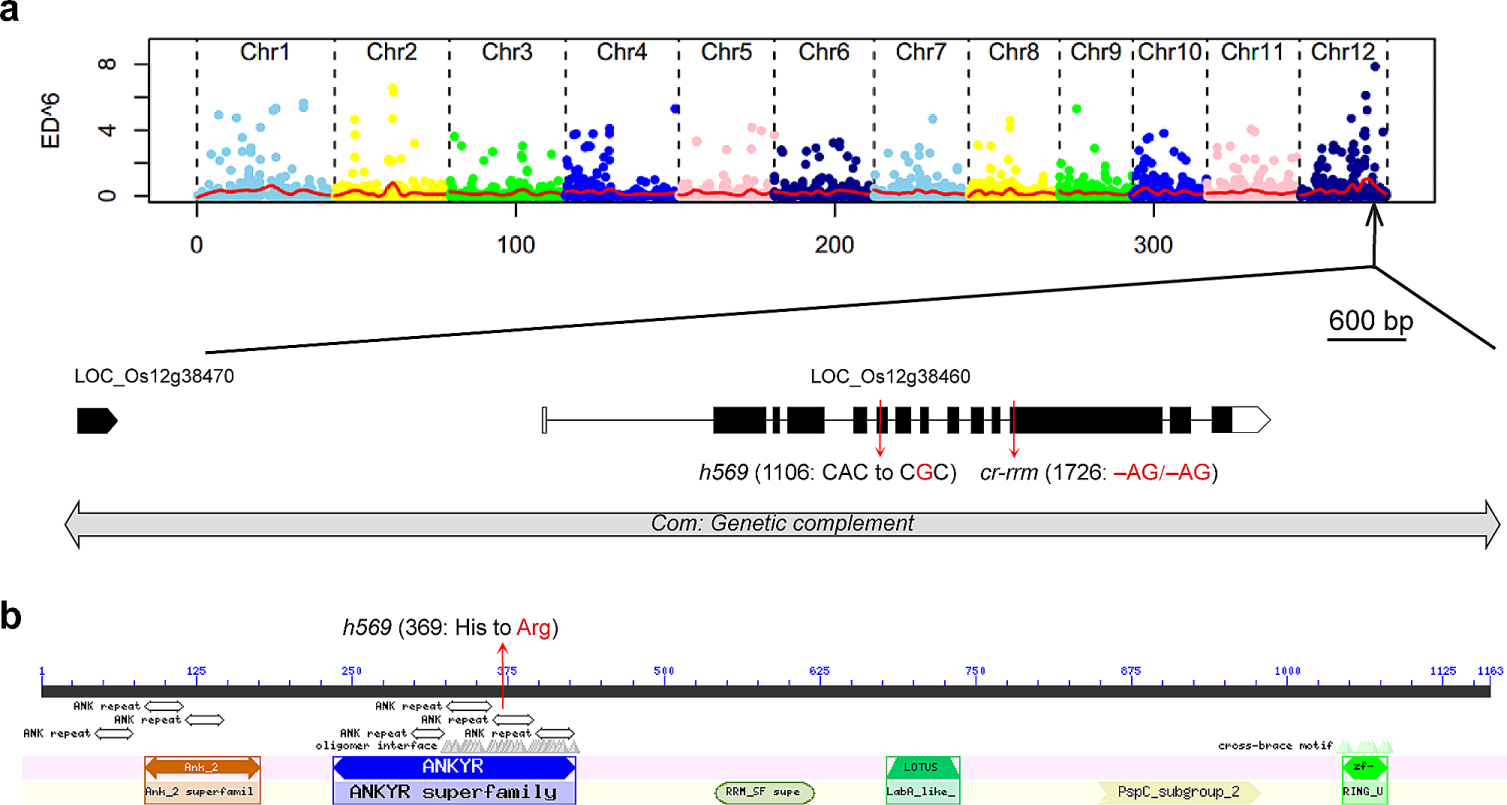
Mutant gene cloning and conserved domain analysis. **a**, Determination of the mutation site. The top section shows the distribution of Euclidean distance (ED) scores of SNP sites along chromosomes in *h569* mutant. *MEL2* (*LOC_Os12g38460*) gene structure with the mutation site in *h569* and the mutation site in the CRISPR knockout mutant *cr-rrm* are shown underneath. White boxes represent 5′- and 3′-UTRs, black boxes represent coding sequence, and the lines between boxes represent introns. The genomic fragment for gene complementation is shown by the double-headed arrow. **b**, Conserved domain analysis of MEL2 protein. Red arrow indicates the amino acid substitution site in the mutant protein in *h569*

The simultaneous identification of multiple causal mutations (SIMM) method described by Yan et al. (2017) was used to define the causal mutation in *h569*. Thirty sterile plants in the F_2_ population were selected for DNA extraction. Equal amount of DNA from each of these plants was pooled and subjected to whole genome resequencing. The sequence data were analyzed using the SIMM pipeline, with the genome sequences of other mutants from the same EMS mutant library as reference (Yan et al. 2017). A candidate mutation (A to G) with the highest mutant index score was identified in the sixth exon of *LOC_Os12g38460*. This A_1106_G mutation caused a codon change from CAC to CGC, resulting in substitution of His_369_ by Arg of the predicted protein (Fig. 3a). To determine the linkage between A_1106_G mutation and phenotype, we genotyped 275 sterile plants and 797 fertile plants in the F_2_ population using high resolution melting (HRM) analysis (Lochlainn et al. 2011). The results showed that all the sterile plants carried the homozygous mutation, whereas the fertile plants showed 2:1 ratio of heterozygous (533) and homozygous WT (264) genotypes, suggesting that the A_1106_G mutation in *LOC_Os12g38460* was likely the causal mutation.

*LOC_Os12g38460* was previously identified as the *MEL2* gene regulating meiosis entry in rice (Nonomura et al. 2011). Conserved domain search indicated that the encoded protein has seven ankyrin (ANK)-repeats at the N-terminus followed by an RNA recognition motif (RRM), a LOTUS domain, a pneumococcal surface protein (PscP), and a zinc finger RING domain (Fig. 3b). The mutation in *h569* is located in the sixth ANK repeat domain (Fig. 3b). The previously reported null mutant of *MEL2* displayed defects in pollen mother cell (PMC) maturation and no pollen (Nonomura et al. 2011), which was completely different from the *h569* mutant phenotype. The null *mel2* mutant also displayed defect in meiosis progression in MMCs (Nonomura et al. 2011), which was similar to the *h569* mutant phenotype.

**Fig. 4:**
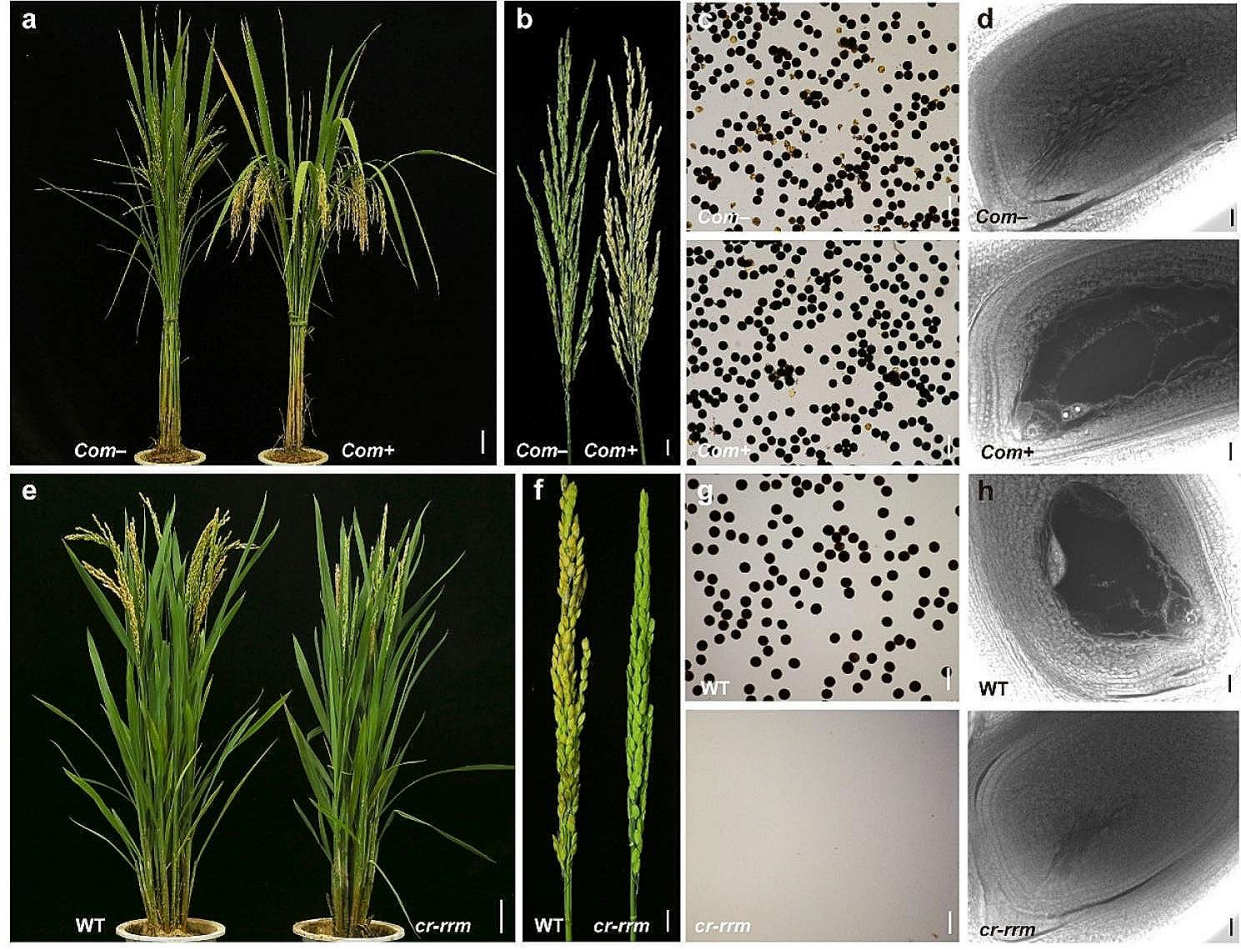
Verification of the mutant gene. **a–d**, Complementation of *h569* mutant. *Com +* and *Com*– represent plants with and without the transgene, respectively. **e–h**, CRISPR knockout of *MEL2* gene in WYG. WT WYG and CRISPR mutant in WYG background (*cr-rrm*) were shown. Seed-setting (a, e), panicles (b, f), I_2_-KI stained pollen grains (c, g), and morphology of embryo sac at mature stage (d, h) were shown. Bars: a, e = 5 cm; b, f = 1 cm; c, g = 100 μm; d, h = 20 μm

To determine if *LOC_Os12g38460* is indeed the mutant gene for *h569*, transgenic complementation was performed with a genomic fragment containing the *LOC_Os12g38460* genic region, 4.1 kb upstream region, and 2 kb downstream sequence (Fig. 3a). The *h569* mutants carrying the transgenic fragment showed normal seed-setting and normal male and female gametophyte development, whereas the uncomplemented mutant still showed the same defects as observed in *h569* mutant (Fig. 4a–d, Table S2), indicating that the A_1106_G mutation in *LOC_Os12g38460* is indeed responsible for the female sterile phenotype. We further performed CRISPR knockout of *LOC_Os12g38460* in Wuyungeng7 (WYG), a *japonica* cultivar easy for transformation. Frame-shift mutants were infertile and showed no pollen grains and defective embryo sac development (Figs. 3a and 4e–h, Table S3). The phenotype of the CRISPR knockout mutants was identical to the previously reported *mel2* mutant (Nonomura et al. 2011), suggesting that the *h569* mutant is likely a partially functional allele that impairs the female meiosis but not the male meiosis.

### Construction of a Female Sterility Maintainer System

The seed production technology (SPT) has been deployed in several plant species for propagation of male sterile lines controlled by recessive nuclear genes (Liao et al. 2021). The strategy is by transforming a male sterile mutant with a T-DNA containing the wild-type fertility restoring gene linked with a pollen-killer gene to specifically kill the transgenic pollen and a seed marker gene to specifically label the transgenic seeds. The resulting transgenic plant can self-pollinate to produce 50% of male sterile seeds and 50% of transgenic seeds that can be distinguished based on the seed marker gene. If the male fertility gene in the T-DNA is replaced by a female fertility gene, and the construct is transformed into the corresponding female sterile mutant, a transgenic maintainer line can be obtained that propagates the female sterile mutant seeds and the transgenic maintainer seeds in 1:1 ratio.

**Fig. 5:**
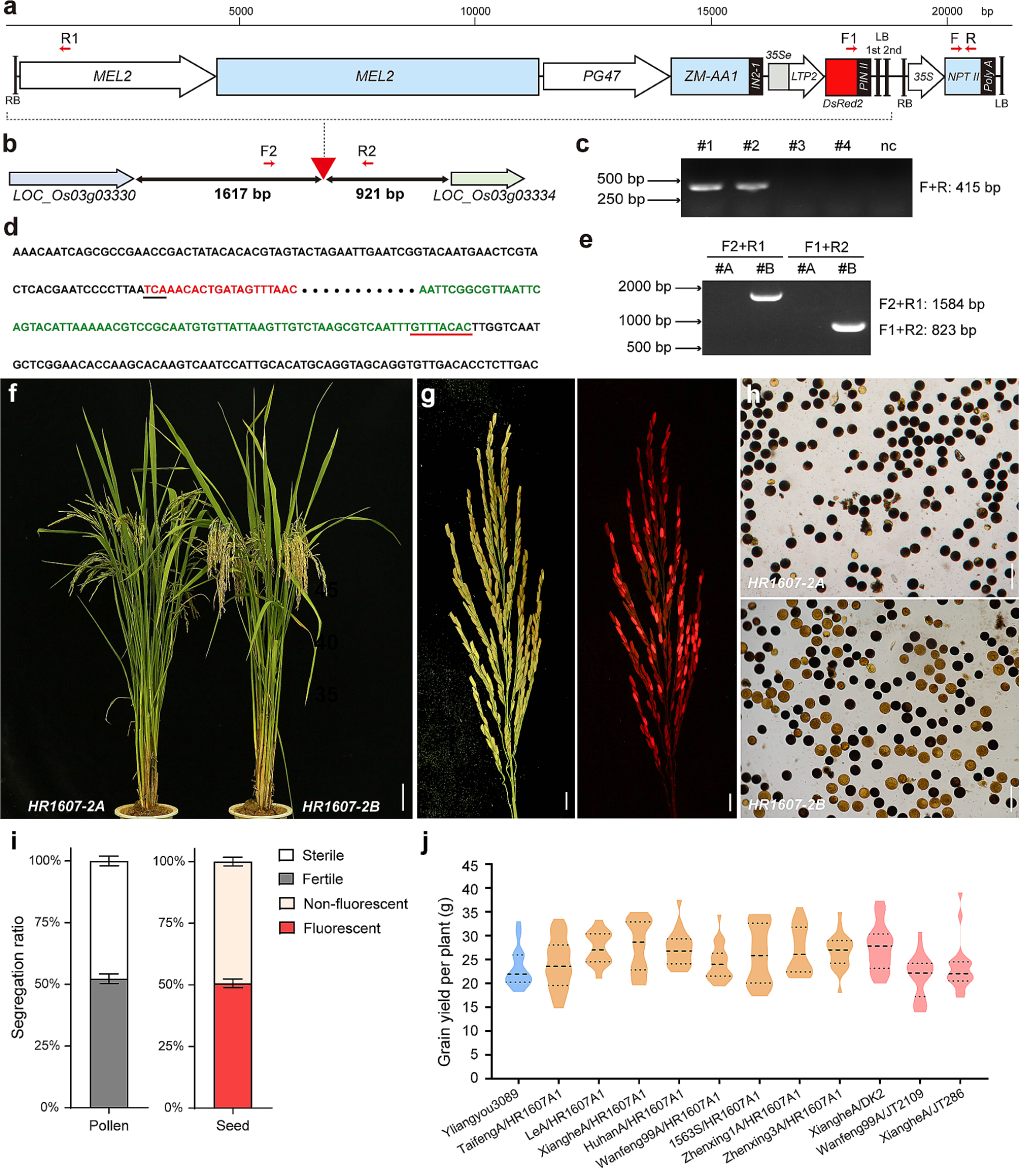
Construction of the female sterility maintainer system. **a**, Diagram of the two T-DNA cassettes in GSX-H569-Red vector. White arrows represent promoters; light blue and red boxes represent genic regions; black boxes represent terminators; and 35Se represents 35S enhancer element. **b**, Diagram showing the T-DNA insertion site in HR1607-2B maintainer genome. Red triangle indicates the insertion site. Double arrows represent the two genes flanking the T-DNA insertion site. The numbers under the line represent the length between the T-DNA insertion site to the two flanking genes. **c**, PCR examination of the transgene. Primers for PCR are as shown in Fig. 5a. #1 and #2 are positive transgenic plants, #3 and #4 are negative transgenic plants, and nc is negative control plant. **d**, Sequence of TAIL PCR products showing the T-DNA insertion site sequence. Sequences marked in black are rice genome sequences flanking the T-DNA insertion site. Sequences marked in red shows the 21 bp right border sequence to the *MEL2* promoter element. Sequence marked in green shows the 76 bp left border sequence to *PIN ΙΙ* element. The right border and left border are underlined in black and red respectively. **e**, PCR verification of the T-DNA insertion site. Primers for PCR are as shown in Fig. 5a. #A indicates HR1607-2A; #B indicates HR1607-2B. **f**, HR1607-2A and HR1607-2B plants at mature stage; bar = 5 cm. **g**, HR1607-2B panicle under bright field (left) and a red fluorescence filter (right); bar = 1 cm. **h**, I_2_-KI staining of the pollen grains of HR1607-2A and HR1607-2B plants; bar = 100 μm. **i**, Rates of fertile and infertile pollen, fluorescent and non-fluorescent seeds produced by HR1607-2B plants. **j**, Yield performance of the F_1_ hybrids. The parental lines for these hybrids are shown in X axis

Because *h569* exhibited a female sterility phenotype, we sought to develop a maintainer system to propagate the *h569* mutant seed. We constructed a double T-DNA binary vector (GSX-H569-Red) containing two T-DNA cassettes (Fig. 5a). The first T-DNA contained the *NPTII* gene under the *35S* promoter for transformation selection. The second T-DNA contained three functional modules: the *MEL2* gene under its native promoter for restoration of female fertility, the maize α-amylase gene *ZmAA1* under the pollen-specific *PG47* promoter to inactivate the transgenic pollen by preventing the formation of starch granules in transgenic pollen, and the red fluorescence protein gene *DsRed2* under the aleurone-specific *LTP2* promoter to make the transgenic seeds producing red fluorescence.

The construct was transformed into Zhongzhonghui1607 (ZZH1607), a widely used restorer line that is easier for transformation. The transgenic lines were examined for the pollen fertility and seed fluorescence. A transgenic line named HR1607-2 displayed approximately 1:1 (2563:2572, χ^2^_1:1_ = 0.016 < χ^2^_0.05, 1_ = 3.84, Table S4) segregation of fertile pollen grains that were darkly stained by I_2_-KI and infertile pollen grains that were lightly stained (Fig. S2a). Self-pollination of HR1607-2 produced approximately 1:1 (140:156, χ^2^_1:1_ = 0.86 < χ^2^_0.05,1_ = 3.84) segregation of fluorscent seeds and non-fluorscent seeds (Fig. S2b). These results indicated that HR1607-2 is a hemizygous transgenic plant containing a single insertion of the second T-DNA.

The fluorescent seeds were germinated and genotyped for the presence of the *NPTII* transgene (Fig. 5c). The plants that did not carry the *NPTII* transgene (named HR1607-2-TD2) were analyzed for the insertion site of the second T-DNA using TAIL-PCR. Sequence analysis of the PCR products indicated that the T-DNA was inserted in the intergenic region between *LOC_Os03g03330* and *LOC_Os03g03334* (Fig. 5b, e). The left and right border sequences of the T-DNA were both present in the TAIL-PCR products (Fig. 5d), indicating that the second T-DNA cassette sequence was complete in the HR1607-2-TD2 plant.

To obtain the maintainer lines that can be used for propagation of the female sterile lines, the HR1607-2-TD2 plant was used as the female parent to cross with *h569* mutant. The resulting F_1_ plants were genotyped for selection of the ones that contained both the T-DNA and the A_1106_G mutation. These F_1_ plants were self-pollinated to set the F_2_ seeds, from which the fluorescent F_2_ seeds were selected and genotyped for the A_1106_G mutation and the transgenes. Because the pollen-killer gene in the T-DNA cassette kills all the pollen grains carrying the transgenes, only the non-transgenic pollen can pass on to the next generation. Therefore, the F_2_ seeds are either transgenes-free or hemizygous for the transgenes. The F_2_ plants with homozygous A_1106_G mutation and hemizygous transgenes were then self-pollinated, and the resulting F_3_ seeds produced by these plants also displayed 1:1 segregation of non-fluorescence and fluorescence (2704:2716, χ^2^_1:1_ = 0.027 < χ^2^_0.05, 1_ = 3.84, Table S5). The non-fluorescent seeds were named HR1607-2A, while the fluorescent seeds were named HR1607-2B.

The HR1607-2A seeds all grew into sterile plants, while the HR1607-2B seeds all grew into fertile plants (Fig. 5f). The seeds produced by the HR1607-2B plant also displayed 1:1 segregation of fluorescence and non-fluorescence (Fig. 5g). I_2_-KI staining experiment indicated that the pollen grains from the HR1607-2A plant were all darkly stained, while the HR1607-2B plant showed 1:1 segregation of darkly stained and lightly stained pollen (Fig. 5h).

### Characteristics of the Female Sterility Maintainer

To investigate if the function of the T-DNA was stable in different genetic backgrounds, we randomly picked 12 HR1607-2B plants for I_2_-KI staining of pollen grains. All of them displayed approximately 1:1 segregation of darkly stained pollen grains and lightly stained pollen grains (Fig. 5i). We randomly picked 24 HR1607-2B plants for observation of seed fluorescence. The seeds of these plants were individually harvested and sorted out based on the fluorescence. All of them showed approximately 1:1 segregation of fluorescent seeds and non-fluorescent seeds (Fig. 5i), suggesting that different genetic background in the F_3_ progeny did not have a significant impact on the T-DNA function. The seeds from these F_3_ generation HR1607-2B plants were harvested separately, sorted out based on fluorescence, and used for analyses of transgene transmission rate and seed-setting rate.

The F_4_ generation HR1607-2B plants all showed normal seed-setting. Approximately 200 F_4_ generation HR1607-2B plants were used as pollen donor to cross-pollinate a male sterile line named Zhenxing1A (ZX1A). A total of 93,370 seeds were obtained from the crosspollination. Among them, 56 seeds were fluorescent, indicating that the transgene transmission rate through the pollen was ~ 0.06%.

The F_4_ generation HR1607-2A plants were sterile or set a few seeds. Because the genetic backgrounds of the F_3_ plants were diverse, we investigated whether the HR1607-2A seeds harvested from different HR1607-2B F_3_ plants had different seed-setting rates. This study was to answer whether the function of A_1106_G mutation is stable in different genetic backgrounds. Table 1 showed that the seed-setting rates ranged from 0 to ~ 0.029% in the different groups of F_4_ plants, suggesting that the difference in genetic backgrounds had little impact on the function of A_1106_G mutation. Because the seed-setting rates of the female infertile lines were extremely low, it suggested that A_1106_G mutation in HR1607-2B can be used for breeding of new maintainer lines of different genetic backgrounds through traditional cross breeding and selection strategy.

**Table 1:** Seed-setting rates of different HR1607-2A lines

Population	TP/SP	Total seeds	TNSPP	SSR
HR1607-2A-4	40/10	35	3000	0.029%
HR1607-2A-6	71/19	28	3100	0.013%
HR1607-2A-8	40/3	4	2300	0.004%
HR1607-2A-10	40/4	5	3000	0.004%
HR1607-2A-12	40/1	2		
HR1607-2A-14	40/1	1		
HR1607-2A-16	40/9	12	3000	0.010%
HR1607-2A-18	40/16	28	2500	0.028%
HR1607-2A-20	40/1	1		
HR1607-2A-22	48/3	3	3200	0.002%
HR1607-2A-24	80/6	7	2490	0.004%
HR1607-2A-28	48/2	2		
HR1607-2A-30	40/1	1		
HR1607-2A-32	40/2	2		
HR1607-2A-34	48/1	1		
HR1607-2A-36	44/3	4	2300	0.004%
HR1607-2A-38	47/2	2		
HR1607-2A-42	40/2	2		
HR1607-2A-44	47/4	6	2400	0.005%
HR1607-2A-50	96/26	29	2000	0.015%
HR1607-2A-26	35/0	0		
HR1607-2A-40	39/0	0		
HR1607-2A-48	39/0	0		

TP/SP: Total number of plants/seeded plants; TNSPP: Total number of spikelets per plant; SSR: Seed-setting rate of population

An F_4_ population named HR1607-A1 with uniform and superior agronomic traits was selected and tested for the potential as male parent. The female sterile plants in this population were crossed with eight different male sterile lines, including five CMS lines, one PTGMS line, and two third-generation male sterile lines. The yield performances of the derived F_1_ hybrids were compared with YLiangyou3089, a local control hybrid variety of Guangdong Province. All eight hybrid rice combinations showed higher yield per plant than the local control (Fig. 5j). HR1607-A1 was also compared with three other paternal lines, DK2, JT2109, and JT286, in test crosses with the commercial CMS lines XiangheA and Wanfeng99A. The XiangheA hybrid with HR1607-A1 showed better yield than the XiangheA hybrids with DK2 and with JT286 (Fig. 5j). The Wanfeng99A hybrid with HR1607-A1 also showed higher yield than the Wanfeng99A hybrid with JT2109. These results indicated that HR1607-A1 holds a good potential to be developed into a valuable male parent line for hybrid rice. However, at present, HR1607-A1 has been bred only to the F_4_ generation, and it is necessary to continue to select excellent individuals until the F_7_ or F_8_ generation to obtain a genetically stable strain.

## Discussion

The breeding and large-scale adoption of hybrid rice is important for modern agriculture and global food supply. However, current hybrid rice production technology depends on extensive labors and is not suitable for mechanized seed production. As a result, the seed production cost is high, and the hybrid rice seed is expensive. This situation is getting worse with the decrease of farming population and increase of labor cost. New technology is urgently needed that can enable mechanized production to compensate for the shortage of manual labor.

Mixed-seeding and mixed-harvesting is the most efficient approach for hybrid rice seed production, which is highly adaptable to mechanization. This approach is possible if the traditional paternal lines are replaced by female infertile lines. To achieve this goal, we sought to develop a technology based on the strategy of the SPT that can be used to propagate the female sterile lines. The principle of SPT is to transform a recessive nuclear male sterile line with the corresponding wild-type male fertility gene linked with a pollen-killer gene and a seed-marker gene (Chang et al. 2016a). The wild-type male fertility gene is to restore the fertility of the mutant plant, the pollen-killer gene is to kill the pollen grains carrying the transgene, and the seed-marker gene is to distinguish the transgenic seeds from the nontransgenic male sterile seeds. A number of systems have been constructed in various crop plants such as maize, rice, and foxtail millet based on this principle, by using various male fertility genes, various pollen killer genes, and different types of seed marker gene (Chang et al. 2016a; Wu et al. 2016, 2021; Zhang et al. 2018, 2023; An et al. 2019; Song et al. 2021; Cai et al. 2023). This principle has also been used for development of a technology aiming at propagation of a female sterile line in rice (Xia et al. 2019). A transgene cassette containing the female fertility restoration gene *POLLEN TUBE BLOCKED 1 (PTB1)*, the pollen inactivation gene *ZmAA1*, and the red fluorescence protein gene *DsRed* was transformed into the *ptb1* mutation background (Xia et al. 2019). The hemizygous *PTB1* transgene fully restored the female fertility of *ptb1* mutant. Self-fertilization of the transgenic plant produced 1:1 *ptb1* mutant seeds and hemizygous transgenic seeds that could be sorted out based on the red fluorescence. However, the *ptb1* mutant is not completely sterile and has a seed-setting rate of 1.8% (Li et al. 2013); this will cause 0.6–1.2% of female infertile seed contamination of the hybrid seed during commercial production (Xia et al. 2019). Li et al. (2022) used a different strategy by constructing a transgene cassette consisting of the wild-type female fertility gene *FST* and the *BTZ-RNAi* expression module, which was then transformed into the female sterile mutant *fst*. The function of *BTZ-RNAi* is to inhibit the expression of *CYP81A6* and make the transgenic plant sensitive to herbicide bentazon. Self-pollination of the hemizygous transgenic T_0_ plants produced 3:1 transgenic and nontransgenic progeny. The transgenic plants were eliminated by bentazon spray, and the transgene-free female sterile line stayed alive (Li et al. 2022). The advantage of this system is that the *fst* mutant is completely female sterile (Li et al. 2022). However, it has a problem in continuing propagation of the maintainer line. Because each generation after self-pollination, the ratio of hemizygous maintainer is reduced while the ratio of homozygous transgenics is increased, and this will eventually exhaust the maintainer line.

Our goal is to develop a maintainer system that can be used for propagation of female sterile lines. To achieve this goal, we started by isolating new female sterile mutant and cloning the mutant gene. Surprisingly, we identified a novel mutation A_1106_G in *MEL2* that conferred female sterility in *h569* mutant. *MEL2* was previously identified by Nonomura et al. (2011) as a gene regulating meiotic entry in both male and female germ cells. Null mutation of *MEL2* abolishes both male and female fertility (Nonomura et al. 2011). MEL2 protein has been shown capable of binding a few meiosis-expressed mRNAs in vitro, but how this activity is related to its function in regulating meiosis entry remains unknown (Miyazaki et al. 2015). MEL2 has seven ANK repeats, an RRM domain, a LOTUS domain, a PscP domain, and a C3HC4 RING E3-ubiquitin ligase domain from N-terminus to C-terminus. This domain combination is present in Poaceae but absent in genomes of Arabidopsis and other organisms (Nonomura et al. 2011). The central region of MEL2 including the RRM domain shows similarities to the human Deleted in Azoospermia (DAZ) Associated Protein 1 (DAZAP1). DAZAP1 is a component of RNA-protein complex that regulates mRNA export from the nucleus to cytoplasm for translation during germ cell development (Moore et al. 2003; Hsu et al. 2008). Another DAZ-interacting protein, DZIP3, has a RING E3-ubiquitin ligase domain (Kreft and Nassal 2003). The LOTUS-containing proteins are germline-specific and are found in the nuage/polar granules of germ cells (Kubíková et al. 2020). These similarities suggest that MEL2 probably has a conserved function in meiosis despite it is specific to monocot plant. ANK repeat domains are involved in protein-protein interactions (Sedgwick and Smerdon, 1999). The *h569* mutant has a point mutation causing an amino acid substitution (His_369_Arg) in the sixth ANK repeat domain. Because this mutation only impairs the female fertility but leaving the male fertility unaffected, we speculate that the seven ANK repeats may function distinctly by interacting with different partner proteins: some partner proteins are specified for meiosis entry of PMCs, while some others are specified for meiosis entry of MMCs. The point mutation in *h569* mutant may disrupt the interaction with protein(s) specified for meiosis entry of MMCs but has little impact on the interactions with protein(s) specific for meiosis entry of PMCs. Further study of the MEL2 interacting proteins, especially those that interact with the ANK repeat domains, will test this hypothesis.

Because *h569* is female infertile but normal in male fertility, we attempted to use this mutant and the wild-type *MEL2* gene to develop maintainer lines that can be used to propagate female sterile paternal lines for hybrid seed production. We constructed the wild-type *MEL2* gene with the pollen killer gene and the seed marker gene into one T-DNA cassette and transformed it into the commercial restorer line ZZH1607. There were two reasons that we chose ZZH1607 for transformation. First, ZZH1607 is an *indica* rice easier for genetic transformation. Second, ZZH1607 is a widely used restorer line that has many superior traits desirable for breeding of new paternal lines. The transgenic plants were first examined for the completeness of the transgene and the transgene insertion site to exclude the plants that carried incomplete transgene or transgene-interruption of a functional rice gene. Growth of the transgenic plants was also compared with nontransgenic plants to exclude those that had growth defects. Transgenic plant that met these selection criteria was crossed with *h569* mutant to bring the transgene and A_1106_G mutation together, and the F_1_ plant was further selfed to produce the F_2_ plants for selection of progeny that was homozygous for the A_1106_G mutation and hemizygous for the transgene. All the selected F_2_ plants displayed the expected 1:1 ratio of fertile and infertile pollen grains and 1:1 female sterile seeds with no red fluorescence and fertile transgenic seeds with red fluorescence, suggesting that the transgene has the expected functions. To breed new paternal lines, self-pollination of the progeny carrying the transgene and homozygous A_1106_G mutation needs be repeated for several generations for selection of progeny of desirable agronomic traits and stable genetic background. By far, the plants are at the F_4_ generation. In each generation, we made careful examinations on the functions of the transgenes, including whether the fertility is completely restored to normal, the proportion of pollen staining, the proportion of fluorescent seeds and nonfluorescent seeds, and the fertility of the plants developed from fluorescent seeds and nonfluorescent seeds. Up to now, abnormal performance of the transgenes has not been found, indicating that the transgenes are functionally stable. The breeding program is still ongoing in our group.

Paternal line of complete female sterility is ideal for mixed-seeding and mixed-harvesting approach. However, *h569* mutant exhibited 0–7 seeds/plant, and the population seed-setting rate was ~ 0.03%. This will lead to 0.01–0.02% female sterile seed contamination of the hybrid seed during mixed harvest. This purity is 200–400-fold higher than the national standard (> 96%) required for hybrid rice seed purity in China and ~ 60-fold higher than the *ptb1*-based system. Because our goal is to use the *mel2*^*A1106G*^ mutation to breed new paternal lines, we need to be certain that the mutation is functionally stable in different genetic backgrounds. We took advantage of the F_2_ plants from ZZH1607 cross *h569* to evaluate the seed-setting rates in different genetic backgrounds. The rates were found to be 0–0.029% in different plants, suggesting that different genetic backgrounds have little impact on the performance of the *mel2*^*A1106G*^mutation.

## Conclusions

Our results indicated that the *mel2*^*A1106G*^ mutation and transgene cassette in HR1607-2B plants can serve the goal of breeding stable maintainer lines for propagation of the female sterile lines. Self-pollination of the maintainer line followed by mechanical seed sorting using a fluorescence seed sorter will be deployed to produce the female infertile line in a large quantity. This technology will reduce the difficulty of multiplying female sterile lines and allows for mechanized seed production using a mixed seeding and mixed harvesting approach. Application of this technology will facilitate mechanization production of hybrid rice seeds and greatly reduce the costs for hybrid rice seed production.

## Materials and Methods

### Plant Material and Growth Conditions

*h569* mutant was isolated from the EMS mutant library derived from the *indica* rice HHZ (Chen et al. 2014). *h569* was crossed with WT HHZ to obtain the F_1_ plant, which was further selfed to produce the F_2_ population. The *japonica* cultivar WYG was used for CRISPR knockout. The *indica* restorer line ZZH1607 was used for transformation of the female sterility maintainer T-DNA construct. All the rice materials were planted in paddy field in Shenzhen with regular care.

### Phenotypic and Genetic Analysis

Rice plants and panicles at yellow mature stage, and spikelets, mature anthers, and mature pistils at the flowering stage were photographed with Canon EOS 5D digital camera or Nikon AZ100 microscope. To examine the fertility of pollen, mature anthers at the flowering stage were crushed in a drop of 1% I_2_-KI solution on a glass slide to release pollen grains. After staining, pollen grains were observed using a Nikon AZ100 microscope and photographed. To determine the female fertility of *h569* mutant, *h569* plants were manually pollinated with the WT HHZ pollen. To determine the male fertility of *h569*, male sterile line ZX1A controlled by recessive nuclear gene *osnp1* was pollinated with the *h569* pollen. Fertility was determined based on the seed-setting of the maternal plants. In the F_2_ population derived from the HHZ and *h569* cross, sterile and fertile plants were distinguished according to their seed-setting phenotype. Statistical analysis of genetic segregation ratio was determined according to the Mendelian inheritance.

### Pollen Germination, Pollen Tube Growth, and Embryonic Sac Observation

Emasculated HHZ and *h569* was artificially pollinated with pollen from the WT HHZ. Two hours after pollination, the pistils of HHZ and *h569* were stained in 0.1% aniline blue solution following the method described by Chang et al. (2016b). Pollen germination and pollen tube growth were observed under a microscope (Nikon AZ100). To observe the ovule development, pistils of WT HHZ and *h569* mutant at various developmental stages were fixed in 70% FAA solution (volume ratio of 70% ethanol, formaldehyde, and acetic acid is 18:1:1), stained with 1% eosin solution, and then observed under a laser confocal microscope (ZEISS LSM800) as described by Wang et al. (2016).

### Identification of the Causal Mutation

The mutation site in *h569* mutant was determined with the SIMM method (Yan et al. 2017). Briefly, the *h569* mutant plant was backcrossed with WT HHZ, and 30 sterile individuals in the F_2_ population were collected and bulk-sequenced. The sequence data were subjected to computational analysis for identification of the mutant gene with the SIMM pipeline as described by Yan et al. (2017). Co-segregation of the candidate mutation with the phenotype in F_2_ population was analyzed using HRM analysis (Lochlainn et al. 2011) with the primer set H569-12g38460HRMF/R (Table S6).

### Plasmid Construction and Rice Transformation

HHZ genomic DNA was used as template for PCR amplification of the *MEL2* genome fragment (including 4.9 kb gene body, 4.1 kb upstream, and 2 kb downstream) with primers 1300-38460-F-EcoRI and 1300-38460-R-HindIII (Table S6). The PCR fragment was cloned into *pCAMBIA1300* binary vector by InFusion recombinant kit (TaKaRa, Dalian, China) to obtain the complementation vector *Com*.

To construct the plasmids for CRISPR knockout of *MEL2* gene, target sequences were designed using the website CRISPR-P v2.0 (http://crispr.hzau.edu.cn/CRISPR2/). The target sequences (Table S6) were cloned into the knockout vector *pYLCRISPR/Cas9-MH* separately following the method of Ma et al. (2015).

To develop the female sterility maintainer line, the dual T-DNA vector *GSX-H569-Red* was constructed. The *pZhen18* vector developed by Chang et al. (2016a) for construction of the maintainer line for propagation of the *osnp1* male sterile mutant was used as the backbone for construction of the *GSX-H569-Red* plasmid. *pZhen18* vector contains two DNAs. The first T-DNA contains the *NPTII* gene driven by the CaMV 35S promoter for transformation selection. The second T-DNA contains the *OsNP1* gene, the pollen killer gene, and the seed marker gene. To replace the *OsNP1* gene with *MEL2* gene, *pZhen18B* plasmid was digested with *Pme* I and *Hin*d III to release the *OsNP1* gene fragment, and the backbone plasmid was saved for further experiment. The *MEL2* genomic DNA was PCR-amplified using the *Com* plasmid as DNA template and 38460-3NGF/38460-11100-R primer pair (Table S6). The PCR fragment was cloned into the *pZhen18B* backbone plasmid using the One Step Seamless Cloning kit (Aidlab Biotechnologies Co., Ltd) to obtain the construct *GSX-H569-Red*.

All the constructs were sequence-verified before transformed into the *Agrobacterium* strain AGL10 for rice transformation.

For complementation experiment, seeds derived the heterozygous mutant were used to develop calli for transformation. Positive transgenic plants are detected by PCR with primers P1 and 1300-38460-2-R (Table S6). The A_1106_G mutation genotype of the positive transgenic plants was confirmed by PCR and HRM analysis using primers 1300-569-PCR-F/R and H569-12g38460HRMF/R (Table S6), respectively.

The CRISPR vectors were transformed into WYG. Positive transgenic plants were identified by PCR using primer SP-L/R (Table S6). Mutation sequence of the *MEL2* gene in the T_0_ positive transgenic plants was determined by PCR amplification of the DNA fragment harboring the target site with primers listed in Table S6, followed by sequencing. The genotypes of the CRISPR target sites in the T_1_ plants were determined by HRM analysis using primers in Table S6.

*GSX-H569-Red* plasmid was transformed into *indica* rice ZZH1607. The positive *NPTII* gene transgenic plants were identified by PCR using primers NPTII-64-415 bp-F (F) and NPTII-64-415 bp-R (R) (Table S6). The *NPTII* positive transgenic plants were further examined for the presence of the maintainer cassette by PCR with the DsRed-F and DsRed-seq-R1 primers. The positive transgenic plants were allowed to self-pollinate, in order to separate the *NPTII* gene and the maintainer cassette in the T_2_ generation. The desired T_2_ transgenic plant lacking the *NPTII* T-DNA cassette but carrying the maintainer cassette (i.e., HR1607-2-TD2) was identified by PCR. The HR1607-2-TD2 plant was analyzed for the segregation ratio of fertile and infertile pollen grains and the segregation ratio of fluorescent and non-fluorescent seeds. The maintainer cassette insertion site was determined by TAIL PCR and sequencing analysis, according to the method described by Wang et al. (2011) and further confirmed by regular PCR and sequencing. The primers for TAIL PCR and regular PCR confirmation are listed in Table S6.

### Characterization of the Female Sterile Maintainer Plant

HR1607-2-TD2 was crossed with *h569* mutant, and the F_1_ plant containing the transgene was self-pollinated to obtain the F_2_ generation. The F_2_ population was genotyped for the A_1106_G mutation by HRM using primers H569-12g38460HRMF/R (Table S6), and the presence of the maintainer T-DNA cassette based on seed color. The F_2_ plants carrying the maintainer T-DNA cassette and homozygous for the A_1106_G mutation were analyzed for pollen fertility using I_2_-KI staining as described above, and the number of darkly stained and lightly stained pollen grains was counted using ImageJ software. The fluorescent and non-fluorescent seeds were sorted using a home-build red fluorescence equipment. Transmission rate of the transgene through pollen was determined by crosspollination of the male sterile line ZX1A with pollen from the maintainer line HR1607-2B. All the seeds grown on the ZX1A plants were harvested for examination of red fluorescence. Transgene transmission rate was determined by the number of fluorescent seeds divided by the number of total seeds. The female sterile plants of the F_4_ generation derived from HR1607-2-TD2 × h569 cross (i.e., HR1607-1A) were tested for yield performance by crossing with available male sterile lines.

### Electronic Supplementary Material

Below is the link to the electronic supplementary material.

Supplementary Material 1

## Data Availability

The datasets supporting the conclusions of this article are included within the article and its additional files.

## Abbreviations

MEL2: meiosis arrested at leptotene2
WT: wild-type
bp: base pair
EMS: ethyl methanesulfonate
HRM: high resolution melting
RRM: RNA recognition motif
DAZ: deleted in Azoospermia
DAZAP: DAZ associated protein
ANK: ankyrin
PMC: pollen mother cell
MMC: megaspore mother cell
