# Supplementary material for: Construction of a Female Sterility Maintaining System Based on a Novel Mutation of the MEL2 Gene
Source: Rice (N Y). 2024 Feb 4;17:12. doi: 10.1186/s12284-024-00688-x (PMC10838886; doi:10.1186/s12284-024-00688-x)
Supplement: Supplementary file 1 — Supplementary Material 1 [file 12284_2024_688_MOESM1_ESM.docx]

**Fig. S1.** Seed-setting rate of *h569* mutant individuals.


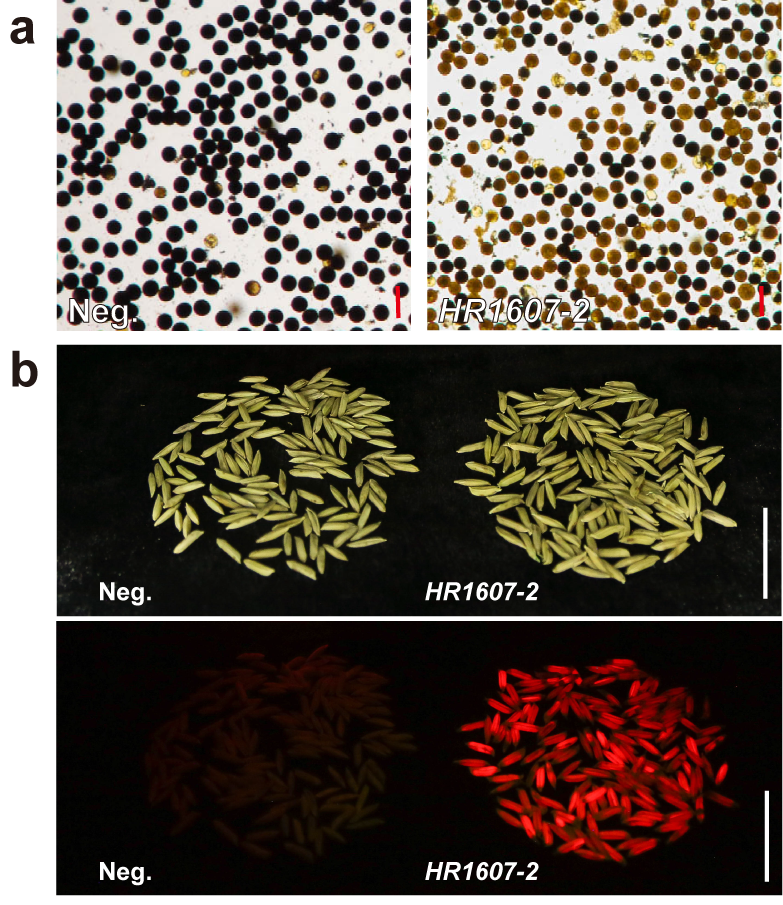


**Fig. S2.** Phenotypes of HR1607-2 transgenic plant. **a,** I_2_-KI staining of the pollen grains of negative transgenic plant (Neg.) and positive transgenic HR1607-2 plants. The functional pollen grains are darkly stained, while the inactivated pollen grains are lightly stained. Bars = 500 μm. **b,** Seeds harvested from the negative transgenic plant (Neg.) and HR1607-2 plant under bright field (top) and a red fluorescence filter (bottom), respectively. Only the seeds carrying the transgene produce red fluorescence. Bar =1 cm.

**Table S1.** The phenotypic separation ratio of the F_2_ progeny.

| Population | Female fertile | Female sterile | Fertile : Sterile (χ^2^_3:1_) |
| --- | --- | --- | --- |
| 1 | 68 | 24 | 0.058 |
| 2 | 70 | 26 | 0.22 |
| 3 | 73 | 22 | 0.17 |
| Total | 211 | 72 | 0.029 |

χ^2^_0.05,_ _1_=3.84

**Table S2.** Genotype and phenotype of the complementation lines.

| Complementation line | Mutation site | Transgenic fragment | Phenotype |
| --- | --- | --- | --- |
| T0-3 | H | + | Fertile |
| T1-3-1 | M | + | Fertile |
| T1-3-2 | W | + | Fertile |
| T1-3-3 | H | – | Fertile |
| T1-3-4 | M | + | Fertile |
| T1-3-5 | W | – | Fertile |
| T1-3-6 | W | – | Fertile |
| T1-3-7 | W | + | Fertile |
| T1-3-8 | H | + | Fertile |
| T1-3-9 | H | + | Fertile |
| T0-4 | M | + | Fertile |
| T1-4-1 | M | + | Fertile |
| T1-4-2 | M | + | Fertile |
| T1-4-3 | M | + | Fertile |
| T0-7 | H | + | Fertile |
| T1-7-1 | H | – | Fertile |
| T1-7-2 | H | – | Fertile |
| T1-7-3 | H | + | Fertile |
| T1-7-4 | W | – | Fertile |
| T1-7-5 | M | – | Sterile |
| T1-7-6 | W | + | Fertile |
| T1-7-7 | W | _ | Fertile |
| T1-7-8 | H | – | Fertile |
| T0-8 | H | + | Fertile |
| T1-8-1 | W | + | Fertile |
| T1-8-2 | H | – | Fertile |
| T1-8-3 | M | + | Fertile |
| T1-8-4 | M | + | Fertile |
| T1-8-5 | H | – | Fertile |
| T1-8-6 | M | – | Sterile |
| T1-8-7 | H | – | Fertile |
| T1-8-8 | W | – | Fertile |
| T1-8-9 | M | – | Sterile |
| T1-8-10 | H | + | Fertile |
| T1-8-11 | H | – | Fertile |
| T1-8-12 | M | + | Fertile |
| T1-8-13 | W | + | Fertile |

W, wild type; H, heterozygous mutation; M, homozygous mutation; +, transgenic positive; –, transgenic negative.

**Table S3.** Genotype and phenotype of the CRISPR knockout mutants.

| Target site | T_0_ line | T_0_ genotype | T_0_ phenotype | T_1_ genotype | Number of T_1_ plants | T_1_ phenotype |
| --- | --- | --- | --- | --- | --- | --- |
| ANK4 | 1CR-ANK4-20 | –AGC/+G | Fertile | –AGC/–AGC | 7 | Fertile |
|  |  |  |  | –AGC/+G | 11 | Fertile |
|  |  |  |  | +G/+G | 6 | Sterile |
|  | 2CR-ANK4-11 | WT/+T | Fertile |  |  |  |
|  | 2CR-ANK4-16 | –AGC/–AGC | Fertile |  |  |  |
|  | 2CR-ANK4-23 | –AGC/–C | Fertile |  |  |  |
| RRM | *cr-rrm* | –AAG/–AG | Fertile | –AAG/–AAG | 12 | Fertile |
|  |  |  |  | –AAG/–AG | 24 | Fertile |
|  |  |  |  | –AG/–AG | 9 | Sterile |
|  | CR-RRM-W2-4 | –AAG/–AAG | Fertile |  |  |  |

**Table S4.** The pollen segregation ratio of HR1607-2 T_0_ plant

| Spikelet | Fertile pollen | Nonfertile pollen | Fertile : Sterile (χ^2^ _1:1_) |
| --- | --- | --- | --- |
| 1 | 964 | 1071 | 5.63 |
| 2 | 657 | 611 | 1.67 |
| 3 | 942 | 890 | 1.48 |
| Total | 2563 | 2572 | 0.016 |

χ^2^_0.05,_ _1_=3.84

**Table S5.** The segregation ratio of F_3_ generation seeds from four HR1607-2-TD2 × *h569* F_2_ plants.

| Plant | Fluorescent seed | Nonfluorescent seed | Fluorescent : Nonfluorescent (χ^2^ _1:1_) |
| --- | --- | --- | --- |
| 1 | 552 | 551 | 0.0009 |
| 2 | 864 | 892 | 0.45 |
| 3 | 619 | 617 | 0.0032 |
| 4 | 669 | 656 | 0.13 |
| Total | 2704 | 2716 | 0.027 |

χ^2^_0.05,_ _1_=3.84

**Table S6.** Primers used in the study.

| **Primers** | **Sequences (5’-3’)** |
| --- | --- |
| ***Com* vector construction and transgenic line detection** | |
| 1300-38460-F-EcoRⅠ | CCATGATTACGAATTCCGCCAACAGTCATGGAATCGTTGATGCTCGAGG |
| 1300-38460-R-HindⅢ | GGCCAGTGCCAAGCTTAGGCAGAGAGGAGGATGAAACTGAAATGGTAGA |
| P1 | ccaggctttacactttatgc |
| 1300-38460-2-R | AGACTAGAGAGGCCCGGTTT |
| 1300-569-PCR-F | CATACTTCTTAAGTTTTTGTGTAGCGTGGCTTG |
| 1300-569-PCR-R | ACTGTCATAATCACCAAAATCTTCCAGGAACTC |
| H569-12g38460HRMF | GCAGGATTGTGTGGCTCCT |
| H569-12g38460HRMR | AAATGCCATCGTGTGAGTAGA |
| **CRISPR vector construction and CRISPR line detection** | |
| CRISPR-RPM-U3-F | GGCAGTAGTCATTGGGATAAGGAT |
| CRISPR-RPM-U3-R | AAACATCCTTATCCCAATGACTAC |
| CRISPR-ANK4-U3-F | GGCAGTACACAGCGCTGCAGCTAA |
| CRISPR-ANK4-U3-R | AAACTTAGCTGCAGCGCTGTGTAC |
| SP-L | GCGCGGTGTCATCTATGTTACT |
| SP-R | CCCGACATAGATGCAATAACTTC |
| CR-ANK/RPM-PCR-F | GCCATGGAAGGTCGTGTTAC |
| CR-ANK/RPM-PCR-R | GGAGCAATTTTCTGGATTTCTGCAG |
| CR-PR/ZF-PCR-F | CATGCGTTCCCTTCCTGGTAT |
| CR-PR/ZF-PCR-R | GCGCAGCAAAATCGAAGGAG |
| ANK4-HRM-F | GGCTCCTGCTGAAGAATGGT |
| ANK4-HRM-R | ACAAAAGGCTACCTGAAGAGC |
| RPM-HRM-F | cctcactgagttcttcactgct |
| RPM-HRM-R | tgagtttcctttgcacttatcacat |
| **GSX-MEL2-Red vector construction and transgenic line detection** | |
| 38460-3NGF | atatcctgtcaaacactgatagtttaaacCGCCAACAGTCATGGAATCGT |
| 38460-11100-R | cgacctgcaggcatgcATATGtAGGCAGAGAGGAGGATGA |
| NPTII-64-415bp-F (F) | CTTGCTCCTGCCGAGAAAGTATCCA |
| NPTII-64-415bp-R (R) | CGTAAAGCACGAGGAAGCGGTCA |
| DsRed-F | atggcctcctccgagaacgtg |
| DsRed-seq-R1 | CTTGTAGATGAAGCAGCCGTCC |
| **Identification and verification second T-DNA insertion site** | |
| TAIL-1A | ccactacctggtggagttcaa |
| TAIL-1B | gccaccacctgttcctgtagttc |
| TAIL-1C | ccagatgcatttcattaaccaaatcc |
| FP1 | GTAATACGACTCACTATAGGGCACGCGTGGT NTCGA STWTS GWGTT |
| FP2 | GTAATACGACTCACTATAGGGCACGCGTGGT NGTCG ASWGA NAWGAA |
| FP3 | GTAATACGACTCACTATAGGGCACGCGTGGT WGTGN AGWAN CANAGA |
| FP4 | GTAATACGACTCACTATAGGGCACGCGTGGT AGWGN AGWAN CAWAGG |
| FP5 | GTAATACGACTCACTATAGGGCACGCGTGGT NGTAW AASGT NTSCA A |
| FP6 | GTAATACGACTCACTATAGGGCACGCGTGGT NGACG ASWGA NAWGAC |
| FP7 | GTAATACGACTCACTATAGGGCACGCGTGGT NGACG ASWGA NAWGAA |
| FP8 | GTAATACGACTCACTATAGGGCACGCGTGGT GTNCG ASWCA NAWGTT |
| FP9 | GTAATACGACTCACTATAGGGCACGCGTGGT NCAGC TWSCT NTSCTT |
| FSP1 | GTAATACGACTCACTATAGGGC |
| FSP2 | ACTATAGGGCACGCGTGGT |
| F2 | CCTCTTATCACTTGTCGGTCATATG |
| R2 | GGCTCTCAGGAATAGGATCGATATG |
| R1 | GCATCAACGATTCCATGACTGTTG |
| F1 | GCCACCACCTGTTCCTGTAGTTC |
